# Intracranial failure after hippocampal-avoidance prophylactic cranial irradiation in limited-stage small-cell lung cancer patients

**DOI:** 10.1038/s41598-021-86851-6

**Published:** 2021-04-01

**Authors:** Yeona Cho, Joongyo Lee, Ik Jae Lee, Jun Won Kim, Jong Geol Baek, Dong Min Jung, Byoung Chul Cho, Min Hee Hong, Hye Ryun Kim, Chang Geol Lee, Hong In Yoon

**Affiliations:** 1 Department of Radiation Oncology, Gangnam Severance Hospital, Yonsei University College of Medicine, Seoul, Republic of Korea; 2 Department of Radiation Oncology, Yonsei Cancer Center, Yonsei University College of Medicine, 50-1 Yonsei-ro, Seodaemun-gu, Seoul, 03722 Republic of Korea; 3 Department of Internal Medicine, Division of Medical Oncology, Yonsei Cancer Center, Yonsei University College of Medicine, Seoul, Republic of Korea

**Keywords:** Small-cell lung cancer, Cancer therapy, Radiotherapy

## Abstract

We evaluated intracranial failure after hippocampus-avoidance-prophylactic cranial irradiation (HA-PCI) for limited-stage small-cell lung cancer (SCLC). Data of 106 patients who received PCI with 25 Gy were retrospectively reviewed. The patients were divided into two groups based on whether they underwent HA-PCI: the HA-PCI group (n = 48) and the conventional PCI (C-PCI) group (n = 58). Twenty-one patients experienced intracranial failure: 11 and 10 patients in the C-PCI and HA-PCI groups, respectively. Using the log-rank test, the intracranial failure rate was not significantly different between the groups (p = 0.215). No clinical factor was significantly associated with intracranial failure in multivariate Cox regression analysis, but HA-PCI tended to be associated with increased incidence of intracranial failure (HR 2.87, 95% CI 0.86–9.58, p = 0.087). Among patients who received HA-PCI, two developed peri-hippocampal recurrence. A higher thoracic radiotherapy dose (≥ 60 Gy) was significantly associated with DFS (HR 0.52, p = 0.048) and OS (HR 0.35, p = 0.003). However, HA-PCI was associated with neither DFS nor OS. Although HA-PCI may be associated with an increased risk of intracranial failure, HA-PCI did not impair disease control or survival. Future prospective randomized trials are needed to reach a definite conclusion.

## Introduction

Small-cell lung cancer (SCLC) is one of the most aggressive types of thoracic malignancy, and its associated outcome is poor. However, survival has recently improved because of advancements in early diagnosis and therapeutic modalities^1,2^. The survival of SCLC patients may be affected by brain metastasis and prophylactic cranial irradiation (PCI), which is the standard treatment for SCLC patients who have a favorable response to their initial treatment^3^. Furthermore, PCI for patients with extensive disease and a good response to chemotherapy increases the overall survival (OS). Therefore, PCI is essential for improving survival and preserving patients’ quality of life^4^. However, a recent Japanese phase III multicenter randomized trial demonstrated that compared with observation, PCI did not result in longer OS in SCLC patients with extensive disease^5^.

There are many concerns and issues regarding administering irradiation to the naive brain, thereby making some physicians and patients reluctant about PCI. Although the irradiation dose in PCI has been reduced, previous studies continued to report that PCI led to significant impairments in short-term memory and cognitive functions^6–8^. Such neurocognitive deficit problems following PCI have led to the consideration of the risks/benefits of PCI and whether PCI meets the fundamental therapeutic goal of preserving the quality of life of cancer patients.

Therefore, strategies to reduce radiation doses to the brain or perform active surveillance have been proposed^9^. Additionally, intensity-modulated radiotherapy (IMRT) and pre-treatment brain MRI allowed the delivery of hippocampal-avoidance (HA)-PCI as an alternative option. Since Ghia et al. reported that only 3.3% of lesions were within 5 mm of the hippocampus compared with 86.4% of lesions within > 15 mm from the hippocampus^10^, HA-whole-brain RT (WBRT) has been widely used for treating brain metastasis. Moreover, a phase II study (RTOG 0933) revealed that HA during WBRT is associated with memory preservation, and a recent prospective study of HA-PCI suggested a benefit of hippocampal sparing in limiting neuropsychological deficits^11,12^. Although the safety and feasibility of HA-PCI have been demonstrated, a risk of recurrence in the spared area has also been reported^10,11,13^.

To date, the specific location of intracranial failure after HA-PCI remains unknown, and improved prediction of the sites of intracranial failure after HA-PCI may allow for HA-PCI to be the standard of care for SCLC. Thus, this study aimed to evaluate the patterns of intracranial failure after HA-PCI in SCLC patients, with a particular focus on the specific site of recurrence with regard to the peri-hippocampal area. We also compared intracranial failure rates between the HA-PCI and conventional PCI (C-PCI) groups.

## Results

### Patients and treatment characteristics

Overall, 106 patients received a radiation dose of 25 Gy in 10 fractions and were included in this analysis. All patients had negative brain MRI results 3 months before the start of PCI or at SCLC diagnosis. Forty-eight patients received HA-PCI using helical tomotherapy (Accuray, Sunnyvale, CA, USA). The remaining 58 patients received conventional PCI (C-PCI) via parallel-opposed lateral portals using a 6-MV photon linear accelerator. Patient and treatment characteristics are described in Table 1.

**Table 1:** Patient and treatment characteristics.

	Total		C-PCI		HA-PCI		p value
	n = 106	%	n = 58	%	n = 48	%	
**Age, years**							
Median	65		65		64		0.424
(Range)	(37–82)		(53–82)		(37–79)		
**Sex**							
Male	93	87.7	54	93.1	39	81.3	0.064
Female	13	12.3	4	6.9	9	18.8	
**ECOG PS**							
0, 1	101	95.3	55	94.8	45	93.8	0.842
2	5	4.7	3	5.2	2	4.2	
**T stage**							
T1	17	16.0	7	12.1	10	20.8	0.127
T2	33	31.1	21	36.2	12	25.0	
T3	25	23.6	10	17.2	15	31.3	
T4	31	29.2	20	34.5	11	22.9	
**N stage**							
N0	12	11.3	8	13.8	4	8.3	0.741
N1	13	12.3	8	13.8	5	10.4	
N2	57	53.8	30	51.7	27	56.3	
N3	24	22.6	12	20.7	12	25.0	
**AJCC stage (7th edition)**							
I	7	6.6	5	8.6	2	4.2	0.796
II	11	10.4	6	10.3	5	10.4	
IIIA	43	40.6	22	37.9	21	43.8	
IIIB	45	42.5	25	43.1	20	41.7	
**Initial treatment**							
RT alone	1	0.9	0	0.0	1	2.1	0.063
Concurrent CRT	98	92.5	51	87.9	47	97.9	
Sequential CRT	4	3.8	4	6.9	0	0.0	
Surgery	3	2.8	3	5.2	0	0.0	
**Chemotherapy regimen**							
None	1	0.9	0	0.0	1	2.1	0.114
EP	102	96.2	55	94.8	47	97.9	
Others	3	2.8	3	5.2	0	0.0	
**Chemotherapy cycles**							
4	69	65.1	43	74.1	26	54.2	0.043
6	36	34.0	15	25.9	21	43.8	
**Thoracic RT dose (Gy)**							
Median	54		54		63		< 0.001
(Range)	(30.6–70.2)		(37.-64.5)		(30.6–72)		
**Thoracic RT fraction size (Gy)**							
Median	2		2		2.1		0.097
(Range)	(1.5–15)		(1.8–15)		(1.5–2.5)		
**Thoracic RT fractions**							
Median	30		28		30		0.036
(Range)	(3–35)		(3–33)		(17–35)		
**Thoracic RT total dose (EQD2)**							
< 60 Gy	38	84.4	5	11.1	43	47.8	< 0.001
≥ 60 Gy	7	15.6	40	88.9	47	52.2	
**Thoracic RT modality**							
3D CRT	52	50.5	49	89.1	3	6.3	< 0.001
IMRT	51	49.5	6	10.9	45	93.8	
**Initial treatment response**							
CR	9	8.5	8	13.8	1	2.1	0.023
PR	94	88.7	47	81.0	47	97.9	
SD	3	2.8	3	5.2	0	0.0	
**PCI modality**							
3D CRT	57	53.8	56	96.6	0	0.0	< 0.001
IMRT	49	46.2	2	3.4	48	100.0	
**Interval, initial treatment to PCI (days)**							
Median	112		125		105		0.003
(Range)	(68–312)		(69–312)		(68–167)		
**Interval, last treatment to PCI (days)**							
Median	28		28		27		0.424
(Range)	(0–146)		(7–146)		(0–116)		
**Treatment period**							
Median	2014		2011		2017		< 0.001
(Range)	(2007–2018)		(2007–2016)		(2015–2018)		

*C-PCI* conventional prophylactic cranial irradiation, *HA-PCI* hippocampus-avoidance PCI, *ECOG PS* Eastern Cooperative Oncology Group Performance Status, *AJCC* American Joint Committee on Cancer, *RT* radiotherapy, *CRT* chemoradiotherapy, *EP* etoposide/cisplatin, *EQD2* Equivalent dose in 2 Gy fractions, α/β = 10, *3D-CRT* 3-dimensional conformal radiotherapy, *IMRT* intensity-modulated radiotherapy, *CR* complete response, *PR* partial response, *SD* stable disease.

The median age of the patients was 65 (range 37–82) years, and most patients (87.7%) were male. Furthermore, most patients (83%) had locally advanced disease with stage IIIA/B according to the 7th edition American Joint Committee on Cancer staging system. In total, 98 patients (92.5%) received concurrent chemoradiation (CRT) as their initial treatment, four received sequential CRT, and three underwent primary surgery. The most commonly used chemotherapy regimen was etoposide/cisplatin in 102 patients (96.2%). Sixty-nine patients received four chemotherapy cycles and 36 received six cycles.

The median radiation dose to primary and/or lymphatic thoracic lesions was 60 Gy (EQD2, Equivalent dose in 2 Gy fractions [EQD2], α/β = 10) in 30 fractions. About half of the patients received IMRT to the lung. The median interval between initial diagnosis and PCI was 112 days, and the median interval between the last treatment and PCI was 28 days.

The 106 patients were divided into two groups based on whether they received HA-PCI: 48 in the HA-PCI group and 58 in the C-PCI group. There were no significant differences in age, sex, performance status, stage, or treatment modality between the two groups. However, more patients in the HA-PCI group compared to the C-PCI group received six chemotherapy cycles (43.8% vs. 25.9%, p = 0.043) and a higher dose of thoracic RT (≥ 60 Gy, 15.6% vs. 88.9%, < 0.001). Patients in the HA-PCI group were treated recently, and IMRT was more commonly used for their treatment; all HA-PCI plans included IMRT, and thoracic RT was also delivered more frequently using IMRT in this group than in the C-PCI group (93.8% vs. 10.9%, p < 0.001). Nevertheless, patients in the C-PCI group showed a higher complete response (CR) rate (13.8% vs. 2.1%, p = 0.023) compared to their initial treatment.

### Intracranial failure and related factors

**Figure 1:**
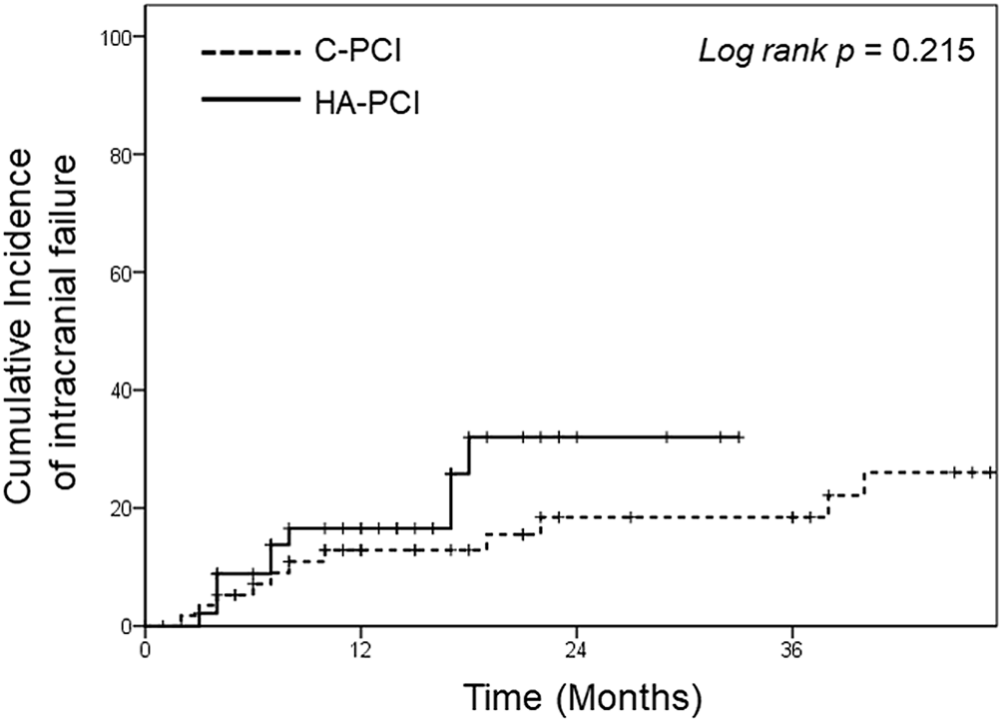
Kaplan–Meir curves of the cumulative incidence of intracranial failure in the conventional prophylactic cranial irradiation (C-PCI) and hippocampal-avoidance (HA)-PCI groups.

The median follow-up duration in all patients was 21 months; it was 18 months in the HA-PCI group and 29 months in the C-PCI group. Twenty-one patients experienced intracranial failure: 11 (19%) in the C-PCI group and 10 (20.8%) in the HA-PCI group. The cumulative incidence of intracranial failure was not significantly different between the two groups (p = 0.215; Fig. 1).

Factors associated with intracranial failure are shown in Table 2. In the univariate analysis, older age and male sex showed a trend toward an increased HR. No clinical factor was significantly associated with intracranial failure in multivariate analysis, but HA-PCI tended to be associated with increased incidence of intracranial failure (HR 2.87, 95% CI 0.86–9.58, p = 0.087).

**Table 2:** Prognostic factors for intracranial failure.

Variables		Univariate analysis				Multivariate analysis			
		HR	95% CI for HR		*p*	HR	95% CI for HR		*p*
			Lower	Upper			Lower	Upper	
Age (years)	≥ 65 (vs. < 65)	0.46	0.19	1.14	0.095	0.6	0.19	1.92	0.391
Sex	F (vs. M)	2.4	0.88	6.58	0.088	1.68	0.47	6.01	0.424
T stage	T3, 4 (vs. T1, 2)	1.12	0.49	2.75	0.737	1.9	0.61	5.92	0.271
N stage	N3 (vs. N0-2)	1.66	0.64	4.33	0.298	1.99	0.54	7.28	0.301
Chemotherapy cycles	6 (vs. 4)	0.78	0.3	2.03	0.61	0.86	0.26	2.8	0.803
Initial treatment response	PR/SD (vs. CR)	0.77	0.23	2.65	0.681	1.4	0.16	12.2	0.761
Interval, diagnosis to PCI (mo)	> 3 (vs. ≤ 3 )	0.91	0.38	2.16	0.827	1.71	0.59	4.97	0.325
Locoregional failure	Yes (vs. No)	1.03	0.44	2.44	0.939	0.81	0.29	2.31	0.697
Hippocampal-avoidance PCI	Yes (vs. No)	1.76	0.71	4.39	0.224	2.87	0.86	9.58	0.087

*RT* radiotherapy, *3D-CRT* 3-dimensional conformal radiotherapy, *IMRT* intensity-modulated radiotherapy, *CR* complete response, *PR* partial response, *SD* stable disease, *PCI* prophylactic cranial irradiation, *HR* hazard ratio, *mo* months.

### Patterns of intracranial failure and salvage treatment

Most patients (8 patients, 80%) who developed brain metastasis after HA-PCI had limited disease with one or two lesions; the remaining patients had multiple brain metastases (more than seven lesions) or leptomeningeal seedings. All patients with intracranial failure received additional brain RT; seven underwent stereotactic radiosurgery (SRS) with 13–16 Gy, two received 30 Gy in 10 fractions of IMRT to all recurrent tumors, and one received 30 Gy of WBRT because of leptomeningeal seedings.

In the C-PCI group, most of patients (9 out of 11 patients, 81.8%) had 1 or 2 brain metastases; of the 11 patients, 6 had SRS, 2 had surgery, 1 had WBRT, and 2 with other distant recurrence had only systemic treatment.

**Figure 2:**
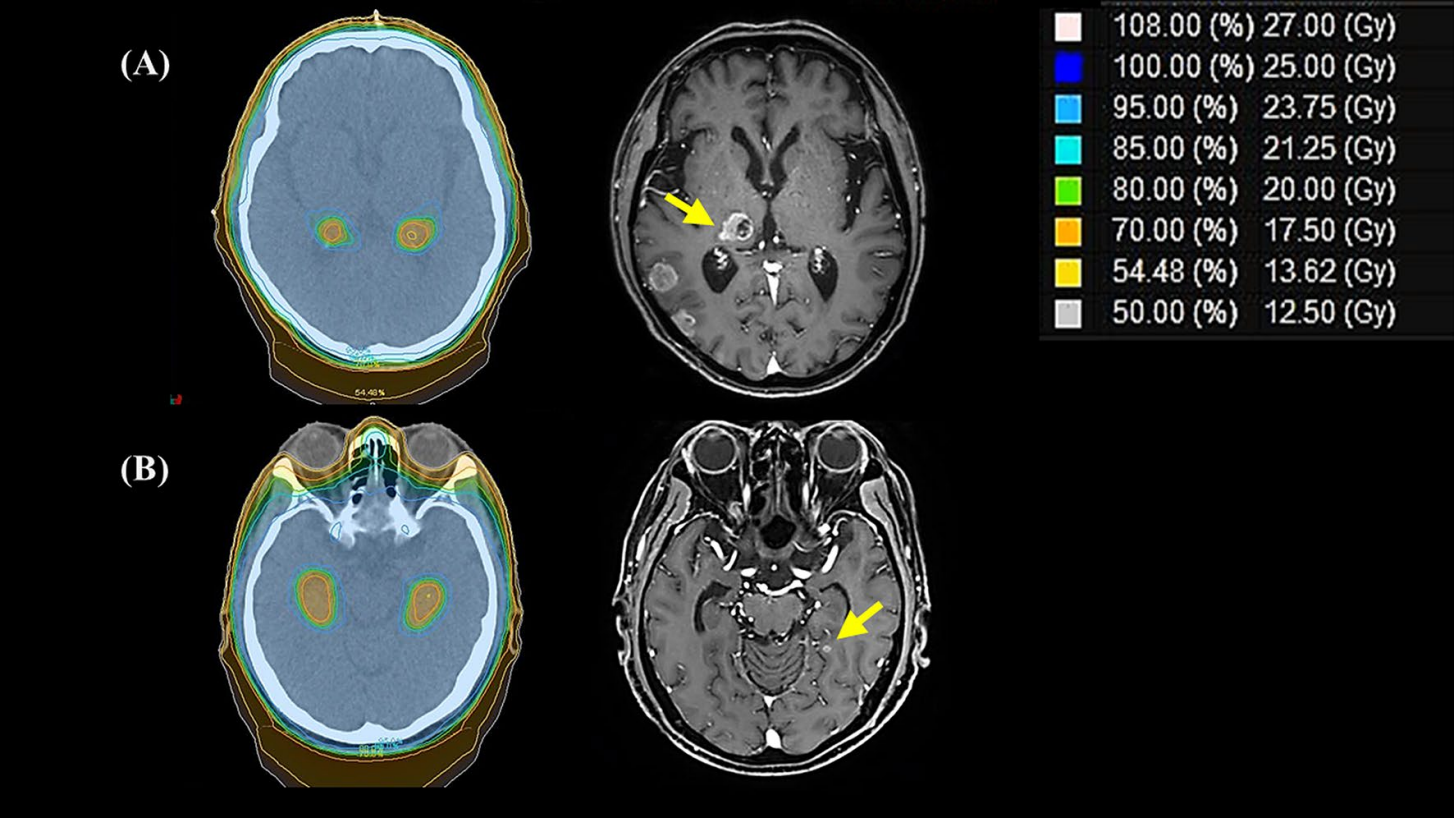
Radiation dose distribution and sites of intracranial failure (ICF) in two patients with peri-hippocampal recurrences after hippocampal-avoidance prophylactic cranial irradiation. Patient (**A**) was a 73-year-old man, and ICF developed 17 months after HA-PCI. Patient (**B**) was a 60-year-old man, and ICF developed 3 months after HA-PCI.

Among patients who received HA-PCI and experienced intracranial failure, two developed brain metastasis in the peri-hippocampal area (Fig. 2), and they developed intracranial failure 17 and 3 months after HA-PCI. Both patients had N3 disease at initial diagnosis, received six chemotherapy cycles, and showed partial response (PR) after the initial treatment. The mean irradiated doses to the peri-hippocampal recurrent tumor were 24.8 and 23.6 Gy, respectively, and the minimum doses were 14.8 and 21.6 Gy, respectively (Fig. 2). In the C-PCI group, only 1 patient showed peri-hippocampal recurrence at multiple other intracranial sites (> 9).

### Disease-free survival and overall survival

**Figure 3:**
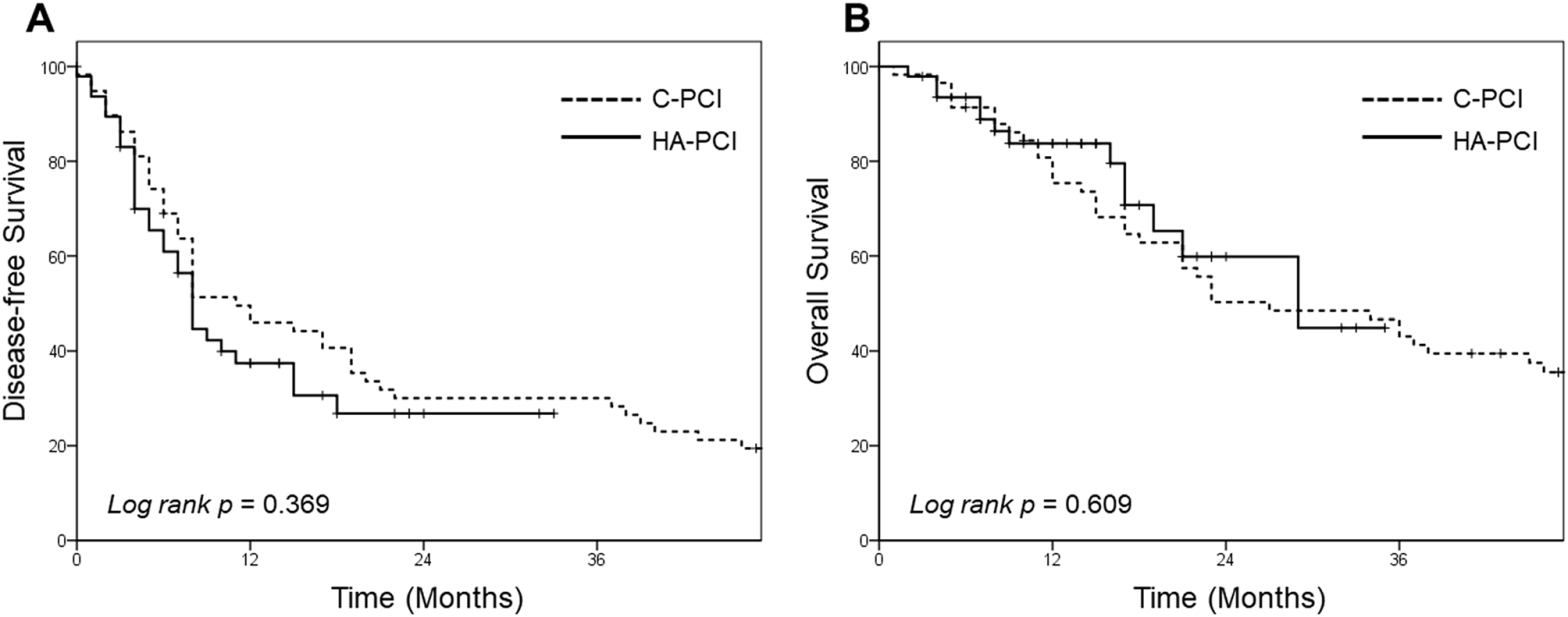
Comparison of disease-free survival (**A**) and overall survival (**B**) between the conventional prophylactic cranial irradiation (C-PCI) and hippocampus-avoidance (HA)-PCI groups.

For all patients, the median disease-free survival [DFS] and OS were 8 and 24 months, respectively. Kaplan-Meir survival curves for the DFS and OS of each group are shown in Fig. 3. Both DFS and OS were not significantly different between groups (C-PCI vs. HA-PCI group: median DFS, 8 vs. 8 months, p = 0.369 and median OS, 23 vs. 24 months, p = 0.609).

Prognostic factors for DFS and OS are shown in Table 3. In the univariate analysis, only a higher thoracic RT dose (equivalent dose in 2 Gy fractions [EQD2] ≥ 60 Gy) was associated with a decreased risk of recurrence (hazard ratio [HR] 0.56, 95% confidence interval [CI] 0.31–0.99, p = 0.049); however, no factor was significant for DFS in multivariate analysis. N stage and thoracic RT dose were associated with OS in the univariate analysis. The multivariate analysis revealed that treatment response showed a trend toward statistical significance for OS (HR 4.04, 95% CI 0.97–17.16, p = 0.058). HA-PCI was not associated with either DFS or OS.

**Table 3:** Results of the Cox regression analyses for disease-free survival and overall survival.

Variables		Disease-free survival						Overall survival					
		UVA			MVA			UVA			MVA		
		HR	95% CI	*p*	HR	95% CI	*p*	HR	95% CI	*p*	HR	95% CI	*p*
Age (years)	≥ 65 (vs. < 65)	0.81	0.52–1.25	0.338	0.6	0.35–1.04	0.067	1.11	0.65–1.85	0.733	0.63	0.31	1.28
Sex	F (vs. M)	0.91	0.46–1.82	0.792	0.56	0.23–1.36	0.197	0.49	0.15–1.58	0.232	0.98	0.12–1.74	0.968
ECOG PS	2 (vs. 0,1)	0.87	0.52–2.91	0.574	1.48	0.52–1.98	0.548	1.21	0.82–2.12	0.312	1.12	0.31–1.87	0.435
T stage	T3, 4 (vs. T1, 2)	1.01	0.65–1.56	0.974	0.87	0.51–1.5	0.625	0.76	0.45–1.29	0.307	0.56	0.28–1.13	0.105
N stage	N3 (vs. N0-2)	1.3	0.78–2.18	0.311	0.92	0.45–1.88	0.819	1.9	1.02–3.54	0.044	1.13	0.42–3.06	0.812
CTx cycles	6 (vs. 4)	1.13	0.71–1.79	0.62	1.15	0.66–2.01	0.616	0.89	0.51–1.58	0.7	1.15	0.58–2.3	0.689
TRT dose (EQD2, Gy)	≥ 60 (vs. < 60)	0.56	0.31–0.99	0.049	1.23	0.6–2.55	0.568	0.32	0.17–0.59	< 0.001	0.95	0.33–2.72	0.921
Treatment response	PR/SD (vs. CR)	1.75	0.8–3.82	0.16	2.37	0.83–6.75	0.107	1.7	0.67–4.27	0.263	4.04	0.95–17.16	0.058
HA-PCI	Yes (vs. No)	1.23	0.77–1.98	0.388	1.13	0.54–2.39	0.741	0.84	0.43–1.64	0.614	0.8	0.26–2.46	0.696

*ECOG PS* Eastern Cooperative Oncology Group Performance Status, *AJCC* American Joint Committee on Cancer, *CTx* chemotherapy, *TRT* Thoracic radiotherapy, *EQD2* Equivalent dose in 2 Gy fractions, α/β = 10, *Fx* fraction, *CR* complete response, *PR* partial response, *SD* stable disease, *HA-PCI* hippocampal-avoidance prophylactic cranial irradiation, *UVA* univariate analysis, *MVA* multivariate analysis, *HR* hazard ratio.

## Discussion

We evaluated the rate and pattern of intracranial failure after HA-PCI in limited-stage SCLC patients. Although two patients experienced peri-hippocampal recurrence after HA-PCI, HA-PCI did not significantly increase the intracranial failure rate. DFS and OS did not significantly differ between HA-PCI and C-PCI.

Although the log-rank test results were not significant (p = 0.215), as shown in Fig. 2, the intracranial failure rate might increase in the HA-PCI group with longer follow-up. However, in two patients with recurrence in the peri-hippocampal area in the HA-PCI group, the lesions occurred at margins, and one of them received a sufficient radiation dose (mean dose, 23.6 Gy; minimum dose, 21.6 Gy)^14^. Therefore, the recurrence could not have been because of HA. Based on our findings, whether HA-PCI increases intracranial failure should be carefully assessed.

Effeney et al. reported that among patients who did not receive PCI, 18.3% developed hippocampal metastasis, resulting in a higher incidence than that in non-SCLC patients^15^; however, previous studies have suggested that HA in SCLC would be safe^16^. Other studies showed that the HA technique reduced neurocognitive dysfunction. A prospective study suggested that hippocampal sparing may be beneficial for limiting the neuropsychological sequelae of brain radiation in SCLC patients^12^. Although two patients (10% of their cohort) developed metastasis in an under-dosed brain region, the tumors were effectively treated with SRS. De Dios et al. also reported that the PCI group showed a significant decline in memory compared to the HA-PCI group^17^. Moreover, a recent randomized phase III trial suggested that there was no significant difference in the incidence of brain metastases between standard PCI and HA-PCI according to their abstract^18^. Although HA-PCI might be associated with an increased risk of brain metastasis in our analysis, it did not interfere with disease control or survival; therefore, to preserve the patients’ quality of life, HA-PCI should be considered. Moreover, most recurrences in the HA-PCI group developed as a limited number of metastases and were treated with SRS and were well-tolerated. In the future, a prospective study should be conducted to assess whether HA-PCI for SCLC increases intracranial failure and neurologic death.

Several Japanese studies recommend close observation rather than PCI for SCLC patients. Ozawa et al. revealed that limited-stage SCLC patients who received PCI showed neither improved OS nor fewer brain metastases^19^. They suggested that PCI was less beneficial if management with MRI and SRS was available. Furthermore, managing patients without PCI could improve outcomes^20^. In a recent randomized phase 3 trial in Japan, compared with observation, PCI did not result in longer OS in SCLC patients with extensive stage disease^5^. However, this approach could instill in patients and physicians’ anxiety and fear of recurrence with limited treatment options. According to the National Comprehensive Cancer Network guidelines, PCI is the standard treatment for both extensive and limited-stage SCLC, and methods of reducing neurocognitive dysfunction within the scope of PCI need to be determined. According to previous dosimetric studies, neurocognitive dysfunction occurs because the mean dose to the hippocampus exceeds approximately 19–20 Gy^21^. Besides HA-PCI, lowering the WBRT dose could be an alternative method. In cases of poor performance status, old age, or extensive stage, low-dose PCI may be useful.

As IMRT was covered by the national insurance program, had better dose coverage of the target volume, and spared OARs, both thoracic and cranial treatments were performed with IMRT. Despite more intensive treatment (i.e., higher thoracic RT doses and frequent use of 6 cycles of chemotherapy), patients in the HA-PCI group showed a lower CR rate than those in the C-PCI group. Although there was no difference in American Joint Committee on Cancer stage between the two groups, the aggressiveness of the tumors might have been different. It might be helpful to evaluate the actual tumor volume or Ki-67 index, which can determine tumor aggressiveness, and other known prognostic markers including serum lactate dehydrogenase and C-reactive protein. However, due to the retrospective nature of this study and long study period, such information was not fully evaluated. This is one of the limitations of this study. Nevertheless, the CR rate was not significant for patient survival, and DFS and OS did not differ between the two groups.

There is a lack of evidence on dose-escalation for SCLC, and 45 Gy in 30 fractions twice daily is the standard regimen^22^. However, several studies suggested the safety and efficacy for dose-escalation for SCLC^23^. In the era of IMRT, administering a higher dose may be recommended, especially for controlling bulky and less-responsive primary tumors. We should thus wait for the results of CALGB 30610-RTOG 0538, a randomized phase III dose-escalation trial for limited-stage SCLC.

This study had several limitations. First, because of the study’s retrospective nature, neurocognitive functions before and after PCI were not evaluated; therefore, we could not demonstrate the benefit of HA-PCI. In addition, it was not possible to clearly identify the schedule of brain metastasis evaluation at each period. Second, the number of events was insufficient to identify a statistically significant variable. Third, since we included patients for a relatively long period (12 years), differences in patient and treatment selection could exist between the two groups. Patients in the HA-PCI group were recently diagnosed and treated. Although recent patients received a higher thoracic RT dose with IMRT, brain metastasis might have been detected earlier owing to a careful follow-up course in these patients.

In conclusion, although HA-PCI may be associated with an increased risk of intracranial failure, all peri-hippocampal recurrences were marginal recurrences, and relatively sufficient doses were administered. Furthermore, HA-PCI did not affect disease control and OS. The results of the prospective randomized phase II/III trial, NRG-CC003, will help to draw definite conclusions.

## Materials and methods

### Study population

All methods were carried out in accordance with relevant guidelines and regulations. The study protocol conformed to the ethical guidelines of the 1975 Declaration of Helsinki, as revised in 1983, and was approved by the Institutional Review Board of Yonsei University Health System (IRB protocol number: 4-2019-0855). The patient records/data were anonymized and de-identified prior to analysis, and informed consent was not obtained from the participants. As this was a retrospective study, it was impossible to proceed with the study without an exemption of consent; this was approved by the IRB on the condition of anonymization of patient’s personal data. Between 2006 and 2018, the medical records of patients diagnosed with limited-stage SCLC without distant metastasis were retrospectively reviewed. Patients who received radiotherapy with or without chemotherapy or surgery and showed a favorable response to initial treatment were candidates for PCI. Patients were excluded if they had previously received overlapping irradiation, brain metastases, other distant metastasis, or a history of another malignancy within the past 5 years.

### Treatment planning

As IMRT has been covered by the national insurance program since 2015, most of the patients received thoracic RT with IMRT; PCI was also conducted with IMRT after 2015. Moreover, as the patients’ quality of life became more important, the preservation of neurocognitive function has become essential in the treatment process. Treatment planning was based on planning computed tomography (CT) without an intravenous contrast agent but with 1–3-mm-thick slice intervals.

For HA-PCI planning, 3D-T1W MRI sequences were fused to their planning CT scans for hippocampal contouring. The anatomic boundaries of the hippocampus were identified according to a previous protocol^10,24^, and based on current recommendations of RTOG 0933, 5-mm volumetric expansion was considered from the hippocampus to create an HA zone^11^. The HA-PCI plan was generated, in which the mean dose to the hippocampus was < 10 Gy and the maximum dose was < 16–17 Gy. Daily mega-voltage CT guidance was also used.

### Follow-up

In general, PCI was performed within 1 month after the end of the last treatment. Before the start of PCI, thoracic disease control was evaluated mainly with chest CT 1–4 weeks after the initial treatment. The response to the initial treatment was evaluated by radiologists using the Response Evaluation Criteria in Solid Tumors criteria and categorized as CR, PR, stable disease, or progressive disease.

After PCI, it was suggested that patients visit the hospital every 3 months for 5 years, and brain MRI was performed at least once a year. If there was evidence of brain metastasis, namely a neurologic symptom, primary tumor progression, or metastasis to another site, additional brain evaluation was performed. For patients who developed brain metastases, T1 post-gadolinium MRI at the time of intracranial failure was manually co-registered into the MIM vista system (MIM Software, Inc, Cleveland, Ohio) for dosimetric data, including the location, irradiation dose, and the volume of each lesion.

### Statistical analysis

Survival and time to recurrence were estimated using the Kaplan–Meier method, and the differences between groups were compared using the log-rank test. Differences in clinical factors between the HA-PCI and C-PCI groups were compared using Fisher’s exact test. Continuous data were compared between the groups using the Mann–Whitney U test. DFS was defined as the time from the date of first treatment to the date of detection of any recurrence, including locoregional, distant, and intracranial failures. OS was defined as the interval from the date of first treatment to death. Univariate and multivariate analyses for intracranial failure, DFS, and OS were conducted using the Cox proportional hazards model. Furthermore, a multivariate Cox hazard regression analysis was performed using all clinical variables used in univariate analysis; the results were reported as HRs with their corresponding 95% CIs. *P* values < 0.05 were considered statistically significant. All analyses were performed using IBM SPSS, version 24.0 (SPSS, Chicago, IL, USA). The datasets generated during and/or analysed during the current study are available from the corresponding author on reasonable request.

## Abbreviations

EQD2: Equivalent dose in 2 Gy fractions, α/β = 10
CI: Confidence interval
CR: Complete response
CRT: Chemoradiation
CT: Computed tomography
C-PCI: Conventional prophylactic cranial irradiation
DFS: Disease-free survival
HA: Hippocampal avoidance
HR: Hazard ratio
MRI: Magnetic resonance imaging
OS: Overall survival
PCI: Prophylactic cranial irradiation
PR: Partial response
RT: Radiotherapy
SCLC: Small-cell lung cancer
SRS: Stereotactic radiosurgery
VMAT: Volumetric-modulated arc therapy
WBRT: Whole-brain radiotherapy

## References

[1] Sone S (2007). CT findings of early-stage small cell lung cancer in a low-dose CT screening programme. Lung Cancer.

[2] Nugent JL (1979). CNS metastases in small cell bronchogenic carcinoma: Increasing frequency and changing pattern with lengthening survival. Cancer.

[3] Ramlov A, Tietze A, Khalil AA, Knap MM (2012). Prophylactic cranial irradiation in patients with small cell lung cancer. A retrospective study of recurrence, survival and morbidity. Lung Cancer.

[4] Slotman B (2007). Prophylactic cranial irradiation in extensive small-cell lung cancer. N. Engl. J. Med..

[5] Takahashi T (2017). Prophylactic cranial irradiation versus observation in patients with extensive-disease small-cell lung cancer: A multicentre, randomised, open-label, phase 3 trial. Lancet Oncol..

[6] Laukkanen E, Klonoff H, Allan B, Graeb D, Murray N (1988). The role of prophylactic brain irradiation in limited stage small cell lung cancer: Clinical, neuropsychologic, and CT sequelae. Int. J. Radiat. Oncol. Biol. Phys..

[7] Volk SA, Mansour RF, Gandara DR, Redmond J (1984). Morbidity in long-term survivors of small cell carcinoma of the lung. Cancer.

[8] Wolfson AH (2011). Primary analysis of a phase II randomized trial Radiation Therapy Oncology Group (RTOG) 0212: Impact of different total doses and schedules of prophylactic cranial irradiation on chronic neurotoxicity and quality of life for patients with limited-disease small-cell lung cancer. Int. J. Radiat. Oncol. Biol. Phys..

[9] Sio TT (2018). The road less traveled: Should we omit prophylactic cranial irradiation for patients with small cell lung cancer?. Clin. Lung Cancer.

[10] Ghia A (2007). Distribution of brain metastases in relation to the hippocampus: Implications for neurocognitive functional preservation. Int. J. Radiat. Oncol. Biol. Phys..

[11] Gondi V (2010). Estimated risk of perihippocampal disease progression after hippocampal avoidance during whole-brain radiotherapy: Safety profile for RTOG 0933. Radiother. Oncol..

[12] Redmond KJ (2017). Prospective study of hippocampal-sparing prophylactic cranial irradiation in limited-stage small cell lung cancer. Int. J. Radiat. Oncol. Biol. Phys..

[13] Marsh JC (2010). Intracranial metastatic disease spares the limbic circuit: A review of 697 metastatic lesions in 107 patients. Int. J. Radiat. Oncol. Biol. Phys..

[14] Meert AP (2001). Prophylactic cranial irradiation in small cell lung cancer: A systematic review of the literature with meta-analysis. BMC Cancer.

[15] Effeney R, Murphy M, Hukins C, Lehman M, Mai G (2018). Risk of hippocampal metastases in small cell lung cancer: Implications for hippocampal sparing cranial irradiation. J. Thorac. Oncol..

[16] Kundapur V, Ellchuk T, Ahmed S, Gondi V (2015). Risk of hippocampal metastases in small cell lung cancer patients at presentation and after cranial irradiation: A safety profile study for hippocampal sparing during prophylactic or therapeutic cranial irradiation. Int. J. Radiat. Oncol. Biol. Phys..

[17] De Dios NR (2019). Phase III trial of prophylactic cranial irradiation with or without hippocampal avoidance for small-cell lung cancer. Int. J. Radiat. Oncol..

[18] Belderbos J (2019). The incidence and location of brain metastases following HA-PCI compared with standard PCI in small cell lung cancer (SCLC): A phase III trial. Int. J. Radiat. Oncol..

[19] Ozawa Y (2015). Management of brain metastasis with magnetic resonance imaging and stereotactic irradiation attenuated benefits of prophylactic cranial irradiation in patients with limited-stage small cell lung cancer. BMC Cancer.

[20] Sakaguchi M, Maebayashi T, Aizawa T, Ishibashi N, Saito T (2016). Treatment outcomes of patients with small cell lung cancer without prophylactic cranial irradiation. J. Thorac. Dis..

[21] Kim KS (2018). Hippocampus-sparing radiotherapy using volumetric modulated arc therapy (VMAT) to the primary brain tumor: The result of dosimetric study and neurocognitive function assessment. Radiat. Oncol..

[22] Faivre-Finn C (2017). Concurrent once-daily versus twice-daily chemoradiotherapy in patients with limited-stage small-cell lung cancer (CONVERT): an open-label, phase 3, randomised, superiority trial. Lancet Oncol..

[23] Zhu L (2016). Increased biological effective dose of radiation correlates with prolonged survival of patients with limited-stage small cell lung cancer: A systematic review. PLoS ONE.

[24] Gondi V (2010). Hippocampal-sparing whole-brain radiotherapy: A "how-to" technique using helical tomotherapy and linear accelerator-based intensity-modulated radiotherapy. Int. J. Radiat. Oncol. Biol. Phys..

